# IFI16 recruits HDAC1 and HDAC2 to deacetylate the Kaposi’s sarcoma-associated herpesvirus (KSHV) latency-associated nuclear antigen (LANA), facilitating latency

**DOI:** 10.1128/jvi.01549-24

**Published:** 2025-02-10

**Authors:** Anandita Ghosh, Jeffrey Britto, Bala Chandran, Arunava Roy

**Affiliations:** 1 Department of Molecular Medicine, Morsani College of Medicine, University of South Florida 685045 , Tampa, Florida, USA; 2 Florida State University College of Medicine 12236 , Tallahassee, Florida, USA; Lerner Research Institute, Cleveland Clinic, Cleveland, Ohio, USA

**Keywords:** Kaposi's sarcoma-associated herpesvirus, LANA, IFI16, histone deacetylase, protein acetylation, promoter binding, human herpesviruses, latency, virus–host interactions, post-translational modification

## Abstract

IFI16 (interferon-γ-inducible protein 16) is an innate-immune DNA sensor that detects viral dsDNA in the nucleus. It also functions as an antiviral restriction factor, playing a crucial role in regulating the latency/lytic balance of several herpesviruses, including Kaposi’s sarcoma-associated herpesvirus (KSHV). We previously demonstrated that IFI16 achieves this by regulating the deposition of H3K9me3 marks on the KSHV genome. Here, we explored whether IFI16 impacts the KSHV latency/lytic balance through additional mechanisms. Our analysis of the IFI16 interactome revealed that IFI16 binds to the class-I HDACs, HDAC1 and HDAC2, and recruits them to the KSHV major latency protein, latency-associated nuclear antigen (LANA). Previous reports have suggested that LANA undergoes lysine acetylation through unknown mechanisms, which results in the loss of its ability to bind to the KSHV transactivator protein (RTA) promoter. However, how the LANA acetylation-deacetylation cycle is orchestrated and what effect this has on KSHV gene expression remains unknown. Here, we demonstrate that LANA, by default, undergoes post-translational acetylation, and during latency, IFI16 interacts with this acetylated LANA and recruits HDAC1/2 to it. This keeps LANA in a deacetylated form, competent in binding and repressing lytic promoters. However, during lytic reactivation, IFI16 is degraded via the proteasomal pathway, leading to the accumulation of acetylated LANA, which cannot bind to the RTA promoter. This results in the de-repression of the RTA and, subsequently, other lytic promoters, driving reactivation. These findings shed new light on the role of IFI16 in KSHV latency and suggest that KSHV utilizes the cellular IFI16-HDAC1/2 interaction to facilitate its latency.

**IMPORTANCE:**

Kaposi’s sarcoma-associated herpesvirus (KSHV) is an oncogenic γ-herpesviruses etiologically associated with several human malignancies, including Kaposi’s sarcoma, primary effusion B-cell lymphoma, and multicentric Castleman’s disease. Understanding the molecular mechanisms governing the establishment and maintenance of latency in γ-herpesviruses is crucial because latency plays a pivotal role in oncogenesis and disease manifestation post-infection. Here, we have elucidated a new mechanism by which IFI16, a previously discovered antiviral restriction factor, is hijacked by KSHV to recruit class-I HDACs on latency-associated nuclear antigen (LANA), resulting in the latter’s deacetylation. The acetylation status of LANA is critical for KSHV latency because it governs LANA’s binding to the KSHV replication and transcription activator (RTA) promoter, an immediate-early gene crucial for lytic reactivation. Depletion of IFI16 results in the accumulation of acetylated LANA, which is incapable of maintaining latency. These newly discovered interactions between IFI16 and LANA and between IFI16 and HDAC1/2 enhance our understanding of KSHV latency regulations.

## INTRODUCTION

Kaposi’s sarcoma-associated herpesvirus (KSHV), or human herpesvirus 8 (HHV-8), is an γ-herpesvirus etiologically associated with a spectrum of human cancers, including Kaposi’s sarcoma (KS), primary effusion B-cell lymphoma (PEL), multicentric Castleman’s disease (MCD), and some forms of osteosarcoma (1–6). Like other herpesviruses, KSHV establishes lifelong latent infections in the nucleus of the host, where the KSHV genomes persist as multiple highly heterochromatinized extrachromosomal episomes. During latency, only a small subset of the KSHV genetic repertoire is expressed, which mainly includes genes of the latency locus that aid in viral episome maintenance, host immune response evasion, and promotion of survival and proliferation of infected cells (7, 8). However, certain physiological stimuli can induce the lytic reactivation of latent episomes, during which the repressed viral heterochromatin transitions into euchromatin, and lytic KSHV genes are expressed, leading to the production of infectious virions and eventual cell death. Two key proteins, LANA (latency-associated nuclear antigen, ORF73) and RTA (replication and transcription activator protein, ORF50), are the major players that drive the balance between latency and lytic cycle. LANA is crucial for the establishment and maintenance of latency, while the expression of RTA is the primary trigger that initiates the switch from latency to lytic reactivation. KSHV, like all other viruses, exploits the cellular machinery of its host at multiple levels to orchestrate its molecular processes.

Interferon-γ-inducible protein 16 (IFI16), a member of the HIN-200 (hematopoietic interferon-inducible nuclear proteins with 200 amino acid repeats) family of cytokines, is a nuclear sensor for foreign double-stranded and single-stranded DNA that plays crucial roles in triggering the inflammasome and the interferon pathways in response to intra-nuclear viral infections such as KSHV, Epstein-Barr virus (EBV), herpes simplex virus (HSV), and HIV (9–13). In addition to its roles as a DNA sensor, IFI16 also plays a critical role in restricting various DNA viruses such as KSHV, HSV-1, EBV, human cytomegalovirus (HCMV), HPV18, and hepatitus B virus (HBV), as well as several RNA and retroviruses, including influenza virus, Chikungunya virus, Zika virus, and HIV-1 (9, 13–24). The primary mechanism by which IFI16 has been identified to restrict these viruses is transcriptional repression of viral gene expression by either epigenetic mechanisms and/or interaction with specific transcription factors at the viral or cellular promoters (25). IFI16’s capacity to interact with and recruit various other cellular proteins is crucial for its antiviral role, and the N-terminal PYRIN domain of IFI16 has been shown to facilitate both homotypic and heterotypic protein–protein interactions (22, 26). We have previously shown that IFI16 restricts KSHV lytic gene transcription and, in effect, plays a vital role in the latency/lytic regulation of KSHV by binding and recruiting the repressive histone H3K9 methyltransferases, SUV39H1, and GLP on the KSHV genome (27, 28). Knockdown (KD) or knockout (KO) of IFI16 resulted in a reduced recruitment of H3K9me3 marks, leading to the de-repression of KSHV lytic loci, which resulted in lytic reactivation (27, 28). However, although H3K9me3 marks are important for KSHV latency, other repressive histone marks, namely, H3K27me3 and H2AK119ub, have also been shown to play crucial roles in the establishment and maintenance of KSHV latency. Therefore, even when the deposition of H3K9me3 marks is perturbed, H3K27me3 and H2AK119ub marks are still deposited on the KSHV genome by the PRC1/2 complexes, which are known to be independent of IFI16 (27). Considering this fact, the degree of lytic reactivation observed upon IFI16 KD (28) appears disproportional to that expected due to the perturbation of H3K9me3 marks alone. This led us to hypothesize that IFI16 possibly has other, yet undiscovered, mechanisms by which it influences the KSHV latent-lytic balance. Here, we investigated this hypothesis and discovered a novel mechanism by which IFI16 regulates the DNA-binding ability of KSHV LANA, leading to the modulation of KSHV latent-lytic balance.

The KSHV LANA (reviewed in references 29–32) is an 1,162-amino-acid protein with an N-terminal proline-rich domain (aa 1-300, NTD) containing a short chromatin-binding domain (CBD), an extensive central internal repeat region (aa 330-919, IR), and a C-terminal domain (aa 931-1162, CTD) containing a DNA-binding domain (DBD). LANA plays multiple roles in latency, including tethering the KSHV episome to the host chromosome, evading the host immune system, modulating the cell cycle, promoting replication of the latent viral genome, driving the stable segregation of viral episome during mitosis, and regulating the transcription of multiple viral and host genes (33–35). LANA’s ability to bind the KSHV genome DNA is essential for many of its functions, such as latent episome replication, episome segregation, and transcriptional regulation. Crystal structures and *in vitro* electrophoretic mobility shift assays (EMSA) have identified the direct binding of LANA via its CTD-DBD to three sequence-specific LANA-binding sites (LBS) on the terminal repeat (TR) region of the KSHV genome: LBS1, 2, and 3 (36–41). These LBSs constitute the latent origins of replication (ori-P), which maintains viral episomal persistence by facilitating its replication once per host cell division. In addition to this sequence-specific DNA binding, LANA also exhibits a sequence-independent DNA-binding mode where it directly interacts with DNA via a positively charged lysine patch in its CTD-DBD (39). This lysine patch is composed of 14 lysine side chains per LANA dimer that are aligned to form a continuous basic surface directly opposite to the sequence-specific DNA-binding surface (39), and has been shown to exert a key role in LANA-mediated KSHV DNA replication and episome persistence (42). Along with these direct interactions with DNA, LANA has also been shown to indirectly bind viral and cellular chromatin via protein–protein interactions with multiple chromatin-associated proteins, including core Histone H2A and H2B, CREB2, mSin3, RING3, MeCP2, SSRP1, and P53 (43, 44).

The ability of LANA to regulate the expression of KSHV lytic genes, particularly the immediate-early (IE) replication and transcription activator protein (RTA, ORF50) gene, is vital for establishing and sustaining KSHV latency (35, 45, 46). Overexpression of LANA lowers RTA expression, while its depletion leads to an increase in RTA mRNA levels in PEL cells latently infected with KSHV (47), indicating its role as a transcription repressor of the ORF50 promoter. LANA has been shown to regulate transcription by binding to the KSHV genome and recruiting specific host transcription and epigenetic factors (48–51). ChIP-seq in PEL cells has revealed that LANA binds to several KSHV promoters, including those of ORFs 8/9, 16, 32/33, 40/42, 50, 57, 71, 73, K7, K8, K14, vIRF2/vIRF3, vIRF1/vIRF4, and the KSHV miRNA cluster (50, 52). However, none of these promoters contain sequences consensus to LBS1/2/3, and how LANA binds the KSHV genome outside of the TR region remains enigmatic. One report suggests that LANA interacts with the transcription repressor, RBP-Jk, and this helps its binding to the RBP-Jk-binding sites 1 to 2 kb upstream of the RTA transcription start site (TSS) (48). However, ChIP-seq data revealed that the LANA binding peak within the RTA promoter is not upstream of the TSS close to the RBP-Jk sites but instead, 600 bp downstream within the ORF50 intron (50). An alternative hypothesis for explaining LANA binding to the KSHV genome outside of the TR is that the C-terminal lysine patch of LANA mediates its sequence-independent binding to KSHV genome DNA. However, this hypothesis has not been experimentally validated.

Interestingly, a 2006 study by Lu et al. showed that LANA’s ability to bind and repress the KSHV RTA promoter depends on its lysine acetylation status (47). They found that HDAC inhibitors such as sodium butyrate (NaB) lead to the rapid dissociation of LANA from the ORF50 promoter, probably due to LANA’s acetylation, disturbing its association with the core histone, H2B, and the transcription factor Sp1, shown to be critical for the regulation of the RTA promoter (53, 54). According to this model, LANA undergoes a cycle of lysine acetylation and deacetylation. When LANA is acetylated, it is unable to bind to the RTA promoter, thus it does not exert any transcriptional pressure. However, when LANA is deacetylated, it can bind to the RTA promoter, leading to its transcriptional repression, which contributes to the establishment or maintenance of latency. Currently, it is unclear how the balance between the acetylated and deacetylated forms of LANA is regulated to favor the prevalence of deacetylated LANA during the establishment and maintenance of latency and to shift this balance toward acetylated LANA upon lytic reactivation.

In this report, in our quest to uncover yet undiscovered mechanisms by which IFI16 regulates KSHV latency, we first identified a novel interaction between IFI16 and the class-I HDACs, HDAC1 and HDAC2. Subsequently, we established that IFI16 also interacts with KSHV LANA and recruits these HDACs to LANA, facilitating its deacetylation, leading to repression of the RTA promoter and eventual latency. Our previous report showed that upon lytic reactivation, IFI16 is degraded via the proteasomal pathway (28). We propose that this depletion of IFI16 during lytic reactivation leads to the disengagement of HDAC1/2 from LANA, resulting in its acetylation and subsequent detachment from the RTA promoter, leading to RTA expression. This study advances our understanding of KSHV latency regulation and uncovers a new role of IFI16 in this process.

## MATERIALS AND METHODS

### Cells

KSHV-positive PEL cell lines BCBL-1 and BC-3, and the KSHV-negative cell line BJAB were obtained from the AIDS Malignancy Consortium (AMC). These cells were cultured in RPMI-1640 medium with GlutaMax (Gibco Life Technologies) and supplemented with 10% (vol/vol) fetal bovine serum (FBS, Atlanta Biologicals) and penicillin–streptomycin (Gibco Life Technologies). Like other B cells, TREX-BCBL-1-RTA cells (55), a kind gift from Dr. Jae Jung, Cleveland Clinic, were also cultured in the abovementioned medium but supplemented with hygromycin B (200 mg/mL). The KSHV latently infected endothelial cell line, TIVE-LTC cells (Telomerase immortalized human umbilical vein endothelial cells with long-term KSHV infection) (56), a kind gift from Dr. Rolf Renne, University of Florida, was cultured in Vascular Cell Basal Medium (ATCC PCS-100-030) supplemented with Microvascular Endothelial Cell Growth kit-VEGF (ATCC PCS-110-041) and 12.5 μg/mL blasticidine. U2OS (wild type [wt]), CRISPR IFI16 KO U2OS clone 67 (55), and HEK293T cells were cultured in Dulbecco’s modified Eagle medium (DMEM, Gibco Life Technologies) supplemented with 10% FBS, penicillin–streptomycin, and 2 mM L-glutamine (Gibco Life Technologies). iSLK and iSLK.219 cell lines (57), a kind gift from Dr. Don Ganem, University of California, San Francisco, were cultured in DMEM supplemented with 10% FBS, G418 (250 μg/mL), and puromycin (1 μg/mL for iSLK, and 5 μg/mL for iSLK.219). We acknowledge that although the SLK cell line was originally believed to be of endothelial origin, it has been found to be contaminated with a renal carcinoma cell line, Caki-1, which is of epithelial origin (58). Nevertheless, this revelation does not impact the conclusions we have drawn from the experiments presented in this work. All cell lines underwent regular testing to ensure they were free of mycoplasma contamination. To study LANA acetylation, cells were treated with 1 mM sodium butyrate (NaB) for 12 h or as indicated.

### KSHV lytic induction, virus production, and *de novo* infection

KSHV lytic cycle was induced in BCBL-1 cells using 20 ng/mL of 12-O-tetradecanoyl phorbol-13-acetate (TPA). Virion production and purification of KSHV were performed according to the previously described protocol (28). The virion copy number was quantified by qPCR using primers specific for the KSHV ORF73 gene following protocols described previously (28). Doxycycline (DOX, 1 mg/mL) was used to induce TREX-BCBL-1-RTA cells. EdU-labeled KSHV was created by tagging KSHV DNA with 10 mM 5-ethynyl-2’-deoxyuridine (EdU) (Thermo Scientific #A10044) dissolved in dimethyl sulfoxide (DMSO). It was then added to the culture medium of TPA-induced BCBL-1 cells. EdU was added to the culture medium twice, once on day 1 and again on day 3 of TPA induction. After day 5, the labeled virus was purified, and the genome copy number was determined, as previously described. For *de novo* KSHV or EdU-KSHV infection, iSLK cells were infected with 30 genome copies per cell in serum-free basal medium for 2 h, then washed with phosphate-buffered saline (PBS) and incubated in a complete medium for 72 h or as indicated.

### Lentivirus-mediated knockdown of IFI16 and HDACs in BCBL1 cells

To knock down our intended proteins, we used human TRC short hairpin RNA (shRNA) constructs (Dharmacon; Horizon Discovery) and co-transfected HEK293T cells along with the lentivirus packaging vectors using the CalPhos mammalian transfection kit (TaKaRa Clontech) using the manufacturer’s protocol. The shRNA constructs used for protein are mentioned below. We used a pool of three to five shRNA clones to avoid off-target effects. As a control, we used pLKO.1 empty vector (Dharmacon; Horizon Discovery). The following shRNA clones were used: IFI16 (TRCN0000019080, TRCN0000019082, TRCN0000019083); HDAC1 (TRCN0000195672, TRCN0000004814, TRCN0000004818, TRCN0000195467, TRCN0000195103); HDAC2 (TRCN0000197086, TRCN0000196590, TRCN0000195198, TRCN0000004823, TRCN0000004819); and HDAC6 (TRCN0000004841, TRCN0000004843, TRCN0000004842, TRCN0000004840, TRCN0000004839). Post-transfection, the culture media were changed after 16 h, and the supernatants containing the packaged lentivirus particles were collected after 48 h and filtered through a 0.45 µm filter. The supernatant of all the clones targeting the same protein was pulled together to transduce the BCBL1 cells in the presence of 5 µg/mL polybrene.

### siRNA-mediated knockdown of IFI16 in iSLK.219 cell

IFI16 was knocked down for adherent cells using human IFI16-specific SMARTpool siRNA (Dharmacon; Horizon Discovery), and non-targeting siRNA (Dharmacon; Horizon Discovery) was used as a negative control. siRNA transfection was performed using the TransIT-X2 Dynamic Delivery System following the manufacturer’s protocol (Mirus Bio). Briefly, opti-MEM containing siRNA (25 nM final concentration) and TransIT-X2 were incubated for 20 min to form the TransIT-X2:siRNA complex and then added dropwise on more than 80% confluent cell plate. Cells were then incubated for 72 h before confirming knockdown.

### LANA and IFI16 overexpression plasmids and transfection in a different cell line

The LANA-overexpressing plasmid pCI-neo full-length LANA (59) and the IFI16-overexpressing plasmid pcDNA3-FLAG-IFI16, Addgene plasmid 35064 (60) were transfected using either the CalPhos mammalian transfection kit (TaKaRa Clontech) or TransIT-X2 Dynamic Transfection Reagent (Mirus Bio), depending on the cell type, following the manufacturer’s protocol.

### Whole cell lysate (WCL) and nuclear and cytoplasmic fractionation

To prepare WCL, cell pellets were lysed using Pierce IP Lysis Buffer (Thermo Fisher Scientific) supplemented with an EDTA-free protease inhibitor cocktail (Selleckchem), phosphatase inhibitor cocktail (Thermo Fisher Scientific), and an HDAC inhibitor cocktail (MCE) for 30 min on ice, followed by sonication at an amplitude setting of 40 for 3 min with pulses of 15 s on and 10 s off on a Qsonica Q700 sonicator. The lysates were then clarified by centrifugation at 13,000 × *g* for 15 min at 4°C. Nuclear and cytoplasmic extracts were prepared using the Nuclear Extract Kit (Active Motif) following the manufacturer’s protocol. The cell lysates were treated with benzonase, an endonuclease (Sigma), for 1 h to eliminate all forms of nucleic acids. Protein concentrations were estimated using the Pierce BCA (bicinchoninic acid) protein assay kit (Thermo Fisher Scientific).

### Western blot (WB)

All immunoblotting experiments resolved equal protein concentrations on 4%–20% Tris-glycine SDS-PAGE gels, except for studying LANA acetylation, where samples were run on 6% Bis-Tris NuPAGE gels. The resolved gels were then blotted onto nitrocellulose membranes on ice at 300 mA for 90 min. The blots were then probed with the respective primary antibodies (Table 1) overnight at 4°C and then probed with horseradish peroxidase (HRP)-conjugated secondary antibodies (Table 1) for detection. The immunoreactive bands were visualized using the Super Signal West Pico chemiluminescent substrate (Thermo Fisher Scientific) and Super Signal West Femto chemiluminescent substrate (Thermo Fisher Scientific). The blots were then visualized on a Bio-Rad XRS+ system and were analyzed using the Bio-Rad Image Lab software.

**TABLE 1:** List of antibodies used in this study

Antibody	Source
β-Actin, anti-rabbit	Proteintech, 20536-1-AP
β-Tubulin, anti-rabbit	Proteintech, 10094-1-AP
IFI16, anti-mouse	Santa Cruz Biotechnology, SC-8023
IFI16, anti-rabbit	Millipore Sigma, HPA073514
KSHV LANA, anti-rat	Millipore Sigma, MABE1109
KSHV LANA, anti-mouse	In-house
KSHV LANA, anti-rabbit	In-house
ASC, anti-mouse	MBL, D086-3
HDAC1, anti-rabbit	Proteintech,10197-1-AP
HDAC1, anti-goat	Origene, TA348968
HDAC2, anti-rabbit	Proteintech, 12922-3-AP
HDAC4, anti-rabbit	Proteintech, 17449-1-AP
HDAC6, anti-rabbit	Proteintech, 12834-1-AP
Control IgG, anti-rabbit	Proteintech, 30000-0-AP
Control IgG, anti-mouse	Abcam, ab18413
Acetyl lysine, anti-mouse	Invitrogen, MA1-2021
FLAG, anti-mouse	Millipore Sigma, B3111
Lamin-B, anti-mouse	Invitrogen, PA5-29121
KSHV gpK8.1a, anti-mouse	In-house
Anti-mouse HRP	Invitrogen, 31430
Anti-rabbit HRP	Invitrogen, 31460
Anti-rat HRP	Invitrogen, 31470
Anti-rabbit IgG Alexa Fluor 488	Invitrogen, A-11008
Anti-mouse IgG Alexa Fluor 488	Invitrogen, A-11001
Anti-rabbit IgG Alexa Fluor 594	Invitrogen, A-11012
Anti-mouse IgG Alexa Fluor 594	Invitrogen, A-11032

### Co-immunoprecipitation (co-IP)

To reduce non-specific binding, cell lysates, prepared in Pierce IP Lysis Buffer (Thermo Fisher Scientific) supplemented with an EDTA-free protease inhibitor cocktail (Selleckchem), phosphatase inhibitor cocktail (Thermo Fisher Scientific), and an HDAC inhibitor cocktail (MCE), were precleaned using a Sepharose protein A and G beads slurry (GE Healthcare Bio-Science) and incubating at 4°C for 30 min. After preclearing, the lysates were incubated with specific antibodies (Table 1) and a mixture of Sepharose protein A and G beads at 4°C overnight. Immunoprecipitated beads were then washed with IP lysis buffer three times and subjected to Western blot analysis. Additionally, for co-IP experiments using anti-rabbit antibodies, we utilized the Dynabeads Protein A Immunoprecipitation kit (Invitrogen).

### Mass spectrometry (MS)

Precleared cell lysates (500 µg) were immunoprecipitated overnight at 4°C with the indicated antibodies (Table 1). Co-IP was performed as described above, and the washed beads were subjected to on-bead trypsin digestion according to standard protocols. The digested peptides were extracted in 50% acetonitrile/0.1% trifluoroacetic acid (TFA) and were characterized using a Thermo Q-exactive-HF-X mass spectrometer coupled to a Thermo Easy nLC 1200. Samples were separated at 300 nL/min on an Acclaim PEPMAP 100 trap (75 μM, 2 cm, c18 3 μm, 100 A) and an easyspray 100 column (75 μm, 25 cm, c18, 100 A) using a 120-min gradient with an initial starting condition of 2% B buffer (0.1% formic acid in 90% acetonitrile) and 98% A buffer (0.1% formic acid in water). Buffer B was increased to 28% over 90 min, then up to 40% in an additional 10 min. High B (90%) was run for 15 min afterward. The mass spectrometer was outfitted with a Thermo nanospray easy source with the following parameters: spray voltage, 1.8; capillary temperature, 250°C; funnel RF level, 40. Parameters for data acquisition were as follows: for MS data, the resolution was 60,000 with an AGC target of 3e6 and a max IT time of 50 ms, the range was set to 400–1,600 *m/z*. MS/MS data were acquired with a resolution of 15,000, an AGC of 1e5, and a max IT of 50 ms, and the top 30 peaks were picked with an isolation window of 1.6 *m/z* with a dynamic execution of 25 s. The resulting data were processed using Max quant 2.0.3.1. Reviewed human and the HHV8 virus databases were downloaded from UniProt and searched with the following parameters: a tryptic enzyme with a max of two missed cleavages, a precursor mass tolerance of 10 ppm, and a fragment mass tolerance of 0.02 Da.

### Immunofluorescence assay (IFA)

Latently infected suspension PEL cells were placed on 10-chamber glass slides and were air dried. After that, the cells were fixed and permeabilized with ice-cold acetone. For adherent cells, cells were grown on 8-well chamber slides coated with poly-lysine (Sigma), fixed with 4% paraformaldehyde for 15 min, and permeabilized with 0.2% Triton X-100 for 20 min. Next, the permeabilized cells were washed with PBS and blocked with Image-iT FX signal enhancer (Invitrogen) for 30 min at 37°C. Subsequently, the cells were incubated with primary antibodies (Table 1) for proteins of interest for 1 h at 37°C, washed, and then incubated with fluorescent dye-conjugated secondary antibodies (Table 1) for 1 h at 37°C. Finally, the slides were mounted using Mounting Medium with DAPI (Sigma) and were observed under a Keyence BZ-X fluorescence microscope at 60× magnification. The images were analyzed using the Keyence analyzer software. All IFA experiments were conducted independently three times, and a representative field is displayed. Colocalization, where indicated, was quantified using the JACoP plugin in ImageJ and is represented as Manders’ coefficients M1 and M2 from the uncropped image. M1 indicates the fraction of channel A that overlaps with channel B, while M2 represents the overlap of channel B with channel A. M1 and M2 values greater than zero indicate positive colocalization, with a value of 1.0 representing 100% colocalization and −1.0 indicating 100% exclusion. The M1 and M2 values are shown in the respective figures.

### Immunofluorescence assay of KS-positive skin tissue

Formalin-fixed, paraffin-embedded deidentified tissue samples from patients with skin KS and healthy individuals were obtained from the ACSR (The AIDS and Cancer Specimen Resource). The slides were washed twice with xylene for 5 min each and then steamed at 95–100°C in Sodium Citrate Buffer (Sigma) for 40 min to retrieve the antigens. Subsequently, the slides were allowed to cool for 20 min and washed with PBST before proceeding with the IFA as previously described.

### Immunofluorescence assay with EdU click chemistry

For EdU staining, the cells were infected with EdU-KSHV (30 copies/cell) for 24 h. The KSHV genome was stained using the Click-iT EdU Alexa Fluor 594 Imaging Kit (Thermo Fisher Scientific) according to the manufacturer’s protocol.

### Proximity ligation assay (PLA)

PLA was carried out using the DUOLink PLA kit from Sigma, following the manufacturer’s instructions. To summarize, cells were grown on 10-well chamber glass slides, then fixed with 4% paraformaldehyde for 15 min and permeabilized with 0.2% Triton X-100 for 20 min. The cells were washed with PBS and treated with Image-iT FX signal enhancer from Life Technologies for 30 min. Next, the slides were incubated with the relevant primary antibodies (Table 1) diluted in DUOLink antibody diluent buffer for 1 h at 37°C. Subsequently, the slides were washed with wash buffer A and incubated with the appropriate species-specific PLA plus and minus probes for 1 h at 37°C. Following this, the slides were washed twice with wash buffer A and then incubated for 30 min with ligation solution before being washed twice with buffer A. The cells were then treated with an amplification-polymerase solution for 100 min at 37°C in a humidified chamber. After washing the slides three times with wash buffer B, they were mounted with a coverslip using a minimal volume of DUOLink *in situ* mounting medium with DAPI. The PLA signals were visualized as distinct fluorescent dots using a Keyence fluorescence microscope at 60× magnification with an oil immersion objective lens.

### Quantitating KSHV gene expression by real-time qRT-PCR

Total RNA was extracted using the RNeasy mini kit (Qiagen), and on-column DNase digestion was performed using an RNase-free DNase set (Qiagen) according to the manufacturer’s protocol. Concentrations of the extracted RNA were estimated using a NanoDrop spectrophotometer. Equal RNA amounts were used to make cDNA using the High-Capacity cDNA reverse transcription kit (Applied Biosystems) using the manufacturer’s protocol. This cDNA was then used as a template for real-time quantitative reverse transcription-PCR (qRT-PCR) using Power SYBR Green PCR Master Mix (Applied Biosystems) on an ABI Prism 7500 detection system (Applied Biosystems). All RNA levels were normalized to the endogenous controls, RNAaseH, and/or β-actin mRNA and were calculated as the delta–delta threshold cycle (ΔΔCT). All primers used have been described previously (25, 28).

### Chromatin immunoprecipitation (ChIP)

For ChIP, nuclei were isolated, and chromatins were sheared using the truChIP Chromatin Shearing kit (Covaris) following the manufacturer’s protocol on a Covaris ME220-focused ultrasonicator. Next, Triton X-100 and NaCl were adjusted in the sheared lysate to final concentrations of 1% and 150 mM, respectively. Shearing efficiency was evaluated using a 2100 Bioanalyzer instrument and the Agilent High Sensitivity DNA Kit (Agilent Technologies) according to the manufacturer’s instruction, ensuring the fragment size was between 200 and 500 bps. Immunoprecipitation (IP) was performed using 10 mg sheared chromatin with 2 mg of desired antibody (Table 1) or ChIP-grade control IgG (Table 1) overnight at 4°C. The chromatin-antibody complex was then incubated with ChIP-grade protein G magnetic beads (Active Motif) for 2 h at 4°C. The immunoprecipitated complex was washed three times with low-salt and once with high-salt wash buffers (Cell Signaling Technology). For chromatin elution, the beads were incubated in ChIP elution buffer (Cell Signaling Technology) at 65°C for 30 min with shaking (1,200 rpm). The eluted chromatin was then incubated with NaCl and proteinase K for 2 h at 65°C to remove all proteins and to reverse cross-linking of the protein–DNA interaction. The ChIP-enriched DNAs were then purified using the ChIP DNA Clean and Concentrator kit (Zymo Research) and were quantitated by qPCR using Power SYBR green PCR master mix (Applied Biosystems) and primers described previously (27). ChIP enrichments were calculated as percent input relative to input chromatin (2%) and were expressed as fold enrichment over the respective control (shC or siC) ChIPs.

## RESULTS

### IFI16 interacts with HDAC1 and HDAC2 in uninfected and KSHV-infected cell lines

**Fig 1:**
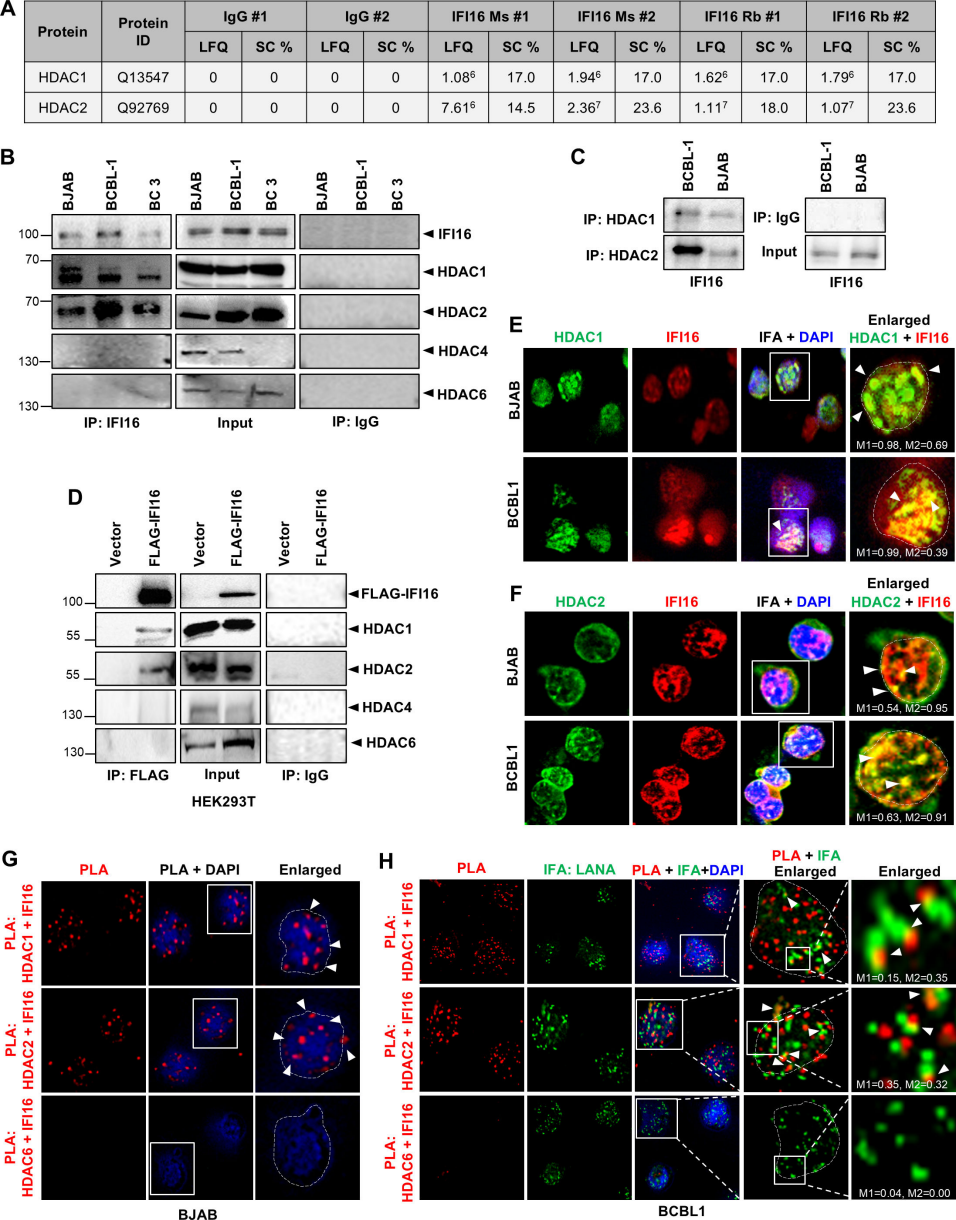
Interaction between IFI16 and HDACs. (**A**) Whole-cell lysates (WCLs) from the KSHV latently infected B-lymphoma cell line, BCBL1, were used for immunoprecipitation (IP) with anti-IFI16 mouse monoclonal (Ms), anti-IFI16 rabbit polyclonal (Rb), or IgG (Rb), followed by purification of the co-precipitated proteins using Protein A/G Dynabeads (two biological replicates, each). Subsequently, on-bead trypsin digestion and LC-MS/MS were performed to identify the co-precipitated proteins. The results include label-free quantitation intensities (LFQ) and sequence coverage (SC %). (**B**) WCLs of KSHV-positive BCBL1, BC3, and KSHV-negative BJAB cells were immunoprecipitated with anti-IFI16 or control IgG antibodies and WB was performed for IFI16, HDAC1, HDAC2, HDAC4, and HDAC6. (**C**) WCLs of BCBL1 and BC3 cells were immunoprecipitated with anti-HDAC1, HDAC2, and control IgG antibodies, and WB for IFI16. (**D**) HEK293T cells, naturally devoid of IFI16 expression, were transfected with a control vector (vector) or FLAG-tagged IFI16-expressing plasmid, immunoprecipitated with anti-FLAG or control IgG antibodies, and WB for the indicated HDACs after 72 h. (**E**) Immunofluorescence assay (IFA) of HDAC1 (green) and IFI16 (red) in BJAB and BCBL1 cells. White arrowheads in the enlarged image point to the colocalization of the two proteins. Manders’ colocalization coefficients, M1 and M2 of the uncropped images are indicated. (**F**) Similar to E, the IFA of HDAC2 (green) and IFI16 (red). Manders’ colocalization coefficients, M1 and M2 of the uncropped images are indicated. (**G**) Proximity ligation assay (PLA, red) was performed for HDAC1, 2, and 6 with IFI16 in BJAB cells. White arrowheads point to PLA dots. (**H**) Similar to G, the PLA (red) of HDAC1, 2, and 6 with IFI16 in BCBL1 cells. Also, the IFA (green) for KSHV LANA was performed in these cells. White arrowheads in the enlarged image point to the colocalization of red PLA dots with green LANA puncta in BCBL1 cells. Manders’ colocalization coefficients, M1 and M2 of the uncropped images are indicated.

To explore whether IFI16 modulates the balance between KSHV latency and lytic phases through mechanisms other than H3K9me3 deposition, we conducted an initial investigation of its interactions with the proteome of cells latently infected with KSHV. For this, we conducted IP using two different anti-IFI16 antibodies, a mouse (Ms) monoclonal and a rabbit (Rb) polyclonal, followed by LC-MS/MS identification of the co-precipitated proteome (*N* = 2) from the lysates of the KSHV latently infected PEL cell line, BCBL-1. We also performed parallel IPs using control IgG (Rb) to detect non-specific interactions. Among the filtered significant positive hits, we observed HDAC1 and HDAC2 as noteworthy interactors of IFI16, which were absent in the IgG pulldowns (Fig. 1A). We then examined whether this IFI16-HDAC1/2 interaction is only specific to cells latently infected with KSHV or is also observable in unrelated, uninfected cell lines. To address this, we analyzed the mass spectrometry data of the IFI16-associated proteome previously published by other research groups. IP of a C-terminal GFP-tagged IFI16 using anti-GFP antibodies from DNase-I-treated HEK293T cell lysates, reported by Diner et al. (61), identified both HDAC1 and HDAC2. In the same report, IP of endogenous IFI16 from THP1 (human leukemia monocytic cell line) cell nuclear lysates also identified HDAC1/2 (61). In another independent report, HDAC1 was identified as an IFI16 interactor in uninfected HFF (human fibroblast) cells (62). However, these reports did not investigate the IFI16–HDAC interaction, and the physiological relevance of this interaction remains unexplored.

Having identified a potential interaction between IFI16 and HDAC1 and 2, we proceeded to confirm this interaction through immunoblotting experiments following co-IP in KSHV latently infected PEL cells (BCBL1 and BC3) as well as the control KSHV and EBV-negative human B-lymphoma cell line, BJAB. We confirmed that this interaction is specific to the class-I HDAC1/2, as HDAC4/6 used as controls did not co-IP with IFI16 (Fig. 1B). We chose HDAC4 and -6 because HDAC4, a class-IIa HDAC, has previously been shown to restrict herpesviruses such as HSV-1 (63), while the ectopic expression of HDAC6, a class-IIb HDAC, has been previously shown to inhibit TPA-stimulated KSHV reactivation independently of HDAC1 (64). Reverse IPs using anti-HDAC1 and HDAC2 antibodies also pulled down IFI16, but its isotype control IgG did not (Fig. 1C). Additionally, we confirmed the specificity of this interaction using FLAG-IFI16 transfected into HEK293T cells, which naturally lack IFI16 expression. Pulldown with anti-FLAG antibody specifically coimmunoprecipitated HDAC1 but not HDAC4/6 in these cells (Fig. 1D). Next, IFA showed that IFI16 colocalizes with HDAC1 and HDAC2 in the nucleus of both uninfected BJAB and latent BCBL-1 cells (Fig. 1E and F).

To further confirm IFI16’s interaction with HDAC1 and -2, we conducted the PLA, which generates a fluorescent signal when the two interacting proteins are within ~40 nm proximity. PLA experiments established that IFI16 interacts with HDAC1 and HDAC2 inside the nucleus of both BJAB and BCBL-1 cells (Fig. 1G and H). Interestingly, when we performed IFA against KSHV LANA in BCBL-1 cells in this experiment, we observed an extensive colocalization (yellow) of LANA puncta (green) with the IFI16-HDAC1/2 PLA signals (red), suggesting that these three proteins are part of the same complex (Fig. 1H). No PLA signal was observed between IFI16 and HDAC6, confirming the specificity of the PLA reaction (Fig. 1G and H). Together, these observations uncover a previously unknown interaction between IFI16 and HDAC1/2 in cells under normal physiological conditions, regardless of their infection status, and propose a potential link between IFI16 and the KSHV latency protein LANA.

### IFI16 interacts with KSHV LANA

**Fig 2:**
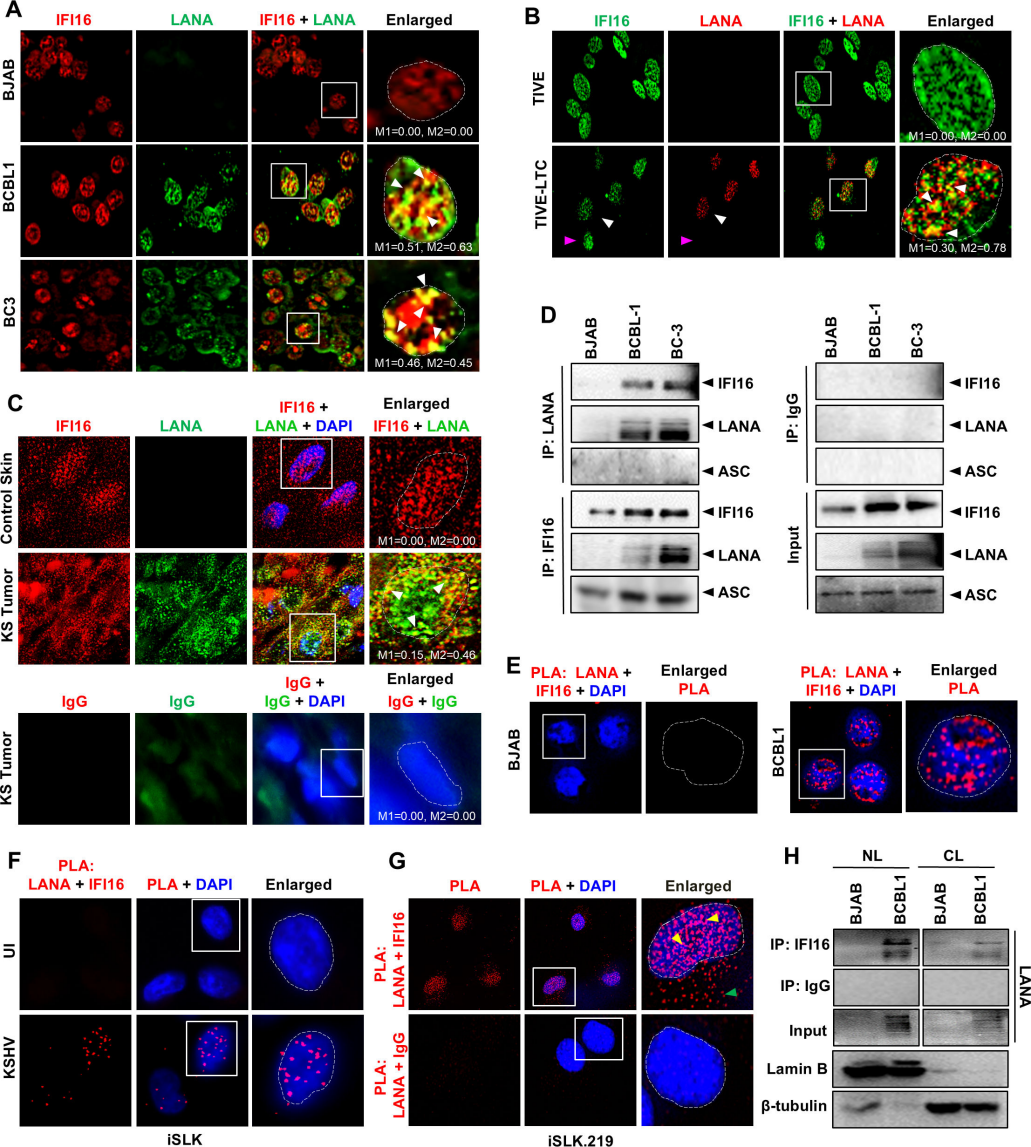
Interaction of IFI16 with the KSHV latency-associated nuclear antigen, LANA. (**A**) IFA of IFI16 (red) and LANA (green) in KSHV-positive BCBL1, BC3, and control BJAB cells. White arrowheads in the enlarged image point to the colocalization of the two proteins. Manders’ colocalization coefficients, M1 and M2 of the uncropped images are indicated. (**B**) IFA of IFI16 (green) and LANA (red) in the KSHV latently infected endothelial cell line, TIVE-LTC, and its uninfected control, TIVE. White arrowheads in the enlarged image point to the colocalization of the two proteins. A fraction of TIVE-LTC cells lack latent KSHV and are thus devoid of LANA expression (pink arrowhead). Manders’ colocalization coefficients, M1 and M2 of the uncropped images are indicated. (**C**) Parafilm-embedded skin tissue samples of healthy subjects (control) and KSHV-infected KS patients were subjected to IFA using anti-IFI16 antibodies (red), anti-LANA Ab (green), and their appropriate IgG controls after antigen retrieval. Colocalization of IFI16 and LANA (yellow) is pointed with white arrowheads. Manders’ colocalization coefficients, M1 and M2 of the uncropped images are indicated. (**D**) WCLs of BJAB, BCBL1, and BC3 cells were immunoprecipitated using anti-LANA, anti-IFI16, and control IgG antibodies, followed by WB for IFI16, LANA, and ASC. (**E**) PLA (red) of LANA with IFI16 in BJAB and BCBL1 cells. (**F**) PLA (red) of LANA with IFI16 in iSLK cells 72 h after *de novo* infection with KSHV (30 DNA copies/cell) or uninfected control. (**G**) PLA (red) of LANA with IFI16 and control IgG in KSHV-positive iSLK.219 cells. Yellow arrowheads in the enlarged image point to the PLA dots inside the nucleus, while green arrowheads point to PLA signals in the cytoplasm. (**H**) Nuclear and cytoplasmic fractions of BJAB and BCBL1 cells were immunoprecipitated using anti-IFI16 and WB for LANA. WB for lamin B and β-tubulin shows the purity of the nuclear and cytoplasmic fractions, respectively.

Next, we investigated the interaction between IFI16 and LANA, and observed substantial colocalization of IFI16 and LANA inside the nucleus of the latently infected PEL cell lines, BCBL1 and BC3 (Fig. 2A), as well as the latently infected endothelial cell line, TIVE-LTC (Fig. 2B). Similar colocalization of IFI16 and LANA was also observed in KS tumor skin biopsies (Fig. 2C). Control skin biopsies lacked LANA staining, which confirmed the specificity of the staining. Notably, IFI16 staining in control biopsies was predominantly nuclear, while, in KSHV-infected tissue, IFI16 was also observable in the cytoplasm (Fig. 2C). This finding aligns with previously published *in vitro* data where we and others showed that following herpesvirus infection, IFI16 undergoes nuclear to cytoplasm redistribution to engage with the interferon and inflammasome cascades (65–68). Next, co-IP experiments confirmed that either protein can pull down the other in latently infected cells (Fig. 2D), whereas IP in uninfected BJAB cells or against control isotype IgG failed to yield similar results. We also confirmed that while IP against IFI16 pulls down ASC, an adaptor protein crucial in forming the inflammasome complex in latently infected cells (9), IP against LANA does not pull down ASC (Fig. 2D). This indicates that the IFI16-LANA complex is independent of the inflammasome complex.

To further investigate the interaction between LANA and IFI16, we performed IFI16-LANA PLA in both KSHV-infected and uninfected B cells. We observed strong nuclear IFI16-LANA PLA signals in BCBL1 cells, confirming the interaction between these two proteins. However, we did not observe these signals in BJAB cells, indicating the specificity of the PLA reaction (Fig. 2E). Similarly, PLA in iSLK cells *de novo* infected with KSHV and in latent iSLK.219 cells also demonstrated strong interactions between IFI16 and LANA in the nucleus (Fig. 2F and G). Interestingly, PLA signals were also observed in the cytoplasm of iSLK.219 cells, which is not surprising and is consistent with previous reports of the presence of cytoplasmic isoforms of both LANA and IFI16 (65, 69). Nuclear-cytoplasmic fractionation also demonstrated that the IFI16–LANA interaction, although predominantly nuclear, is also observable in the cytoplasm of BCBL1 PEL cells (Fig. 2H). Together, these observations show that IFI16 and LANA interact with each other in latently or *de novo* infected cells, and this interaction is predominant in the nucleus.

### IFI16 and LANA can interact in the absence of KSHV genome DNA

**Fig 3:**
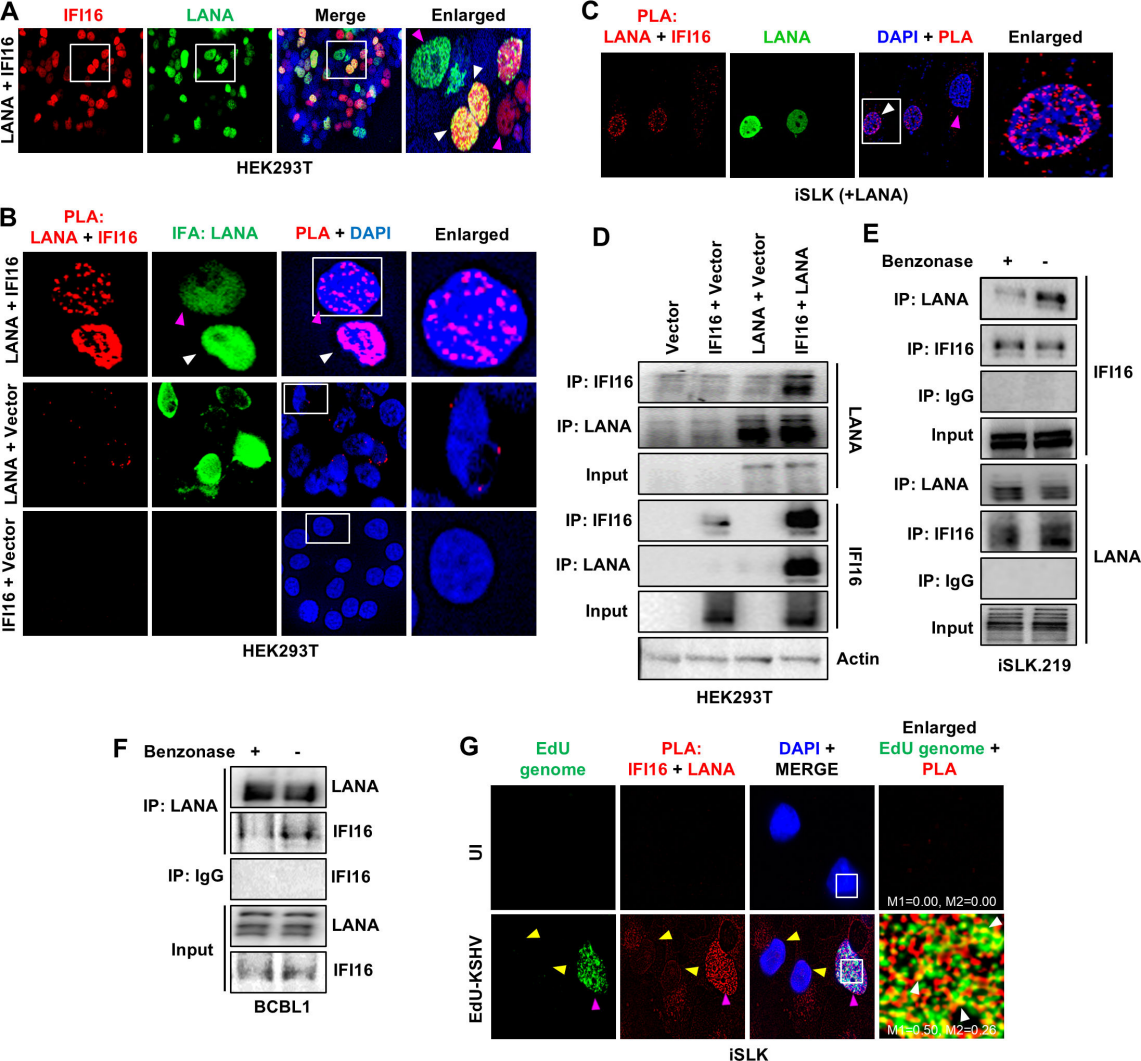
Role of the KSHV genome DNA in IFI16–LANA interaction. (**A**) IFA of IFI16 (red) and LANA (green) in HEK293T cells, naturally deficient in IFI16 expression, transfected with plasmids expressing IFI16 and LANA. White arrowheads point to the colocalization of IFI16 and LANA (yellow) in cells expressing both proteins, while pink arrowheads point to cells expressing only IFI16 or only LANA. (**B**) PLA of LANA and IFI16 (red) together with LANA IFA (green) in HEK293T cells transfected with IFI16 and LANA. LANA + vector and IFI16 + vector-transfected cells are used to show the specificity of the PLA reaction. (**C**) Similar to (**B**) but in KSHV-negative iSLK cells transfected with LANA only. The white arrowhead points to a cell that has been successfully transfected with LANA, while the pink arrowhead points to a cell that has not. (**D**) WCLs of HEK293T cells transfected with the indicated plasmids wereimmunoprecipitated with anti-IFI16 and anti-LANA antibodies, and WB for the indicated proteins. (**E**) WCLs of KSHV(+) iSLK.219 cells were incubated with and without benzonase for 10 min at room temperature and then immunoprecipitated with anti-LANA, anti-IFI16, and IgG control antibodies, followed by WB for the indicated proteins. (**F**) Similar to (**E**), WCL of BCBL1 was incubated with or without benzonase for 10 mins before IP with anti-LANA and anti-IgG antibodies, and WB for IFI16 and LANA. (**G**) PLA of IFI16 and LANA (red) in iSLK cells infected with EdU-labeled KSHV (30 DNA copies) for 72 h. The pink arrowhead indicates a cell infected with EdU-KSHV, while uninfected cells are indicated with yellow arrowheads. Click-iT EdU Alexa Fluor 594 Imaging Kit (green) was used to stain the KSHV genome. Colocalization of red (PLA) with green (EdU-KSHV genome) resulting in yellow (white arrowheads, enlarged panel) indicates the interaction of IFI16 with LANA on the KSHV genome. Manders’ colocalization coefficients, M1 and M2 of the uncropped images are indicated.

IFI16 is a DNA sensor that recognizes foreign DNA and activates the innate immune pathways (9, 16, 17, 67). IFI16 is also known to be associated with the latent KSHV genome during prolonged latency (28, 70). On the other hand, one of the primary functions of LANA is to tether the KSHV genome to the host chromosome. Thus, both IFI16 and LANA have functions where they interact with the KSHV genome DNA. We, therefore, asked whether the observed interaction between IFI16 and LANA is due to their mutual interaction with the same KSHV DNA. To address this, we first studied their colocalization in HEK293T cells transfected with plasmids expressing both proteins. A robust colocalization was observed in this KSHV genome-free system (Fig. 3A). PLA experiments in HEK293T cells further confirmed that these two proteins interact in the absence of KSHV DNA (Fig. 3B). The PLA signals were found to be proportional to the amount of LANA expression (IFA, green) in the transfected cell. As shown in Figure 3B, the cell marked by the white arrowhead, which has higher levels of LANA expression, exhibits a greater PLA signal compared to the cell marked by the pink arrowhead, which has lower levels of LANA expression. A similar PLA was also observed between endogenous IFI16 and transfected LANA in uninfected iSLK cells (Fig. 3C). Pulldown of IFI16 or LANA from HEK293T cells transfected with IFI16 and LANA, or their appropriate vector controls, yielded each other as a coimmunoprecipitated interactor (Fig. 3D). A similar co-IP was also observed after treating lysates of iSLK.219 and BCBL1 with benzonase, a pan-DNA/RNA endonuclease (Fig. 3E and F, respectively). However, a reduction in IFI16–LANA interaction was observed after benzonase treatment, suggesting a potential role of KSHV genome DNA in stabilizing this complex further.

Next, having demonstrated that the IFI16-LANA complex can form independently of the KSHV genome, we asked if this complex interacts with the KSHV genome after its formation. For this, we infected iSLK cells with EdU-labeled KSHV and performed tandem IFI16-LANA PLA and EdU genome staining by click chemistry. Our observations showed that while some of the IFI16-LANA PLA dots remain unassociated with KSHV DNA, some do colocalize with KSHV genomes (Fig. 3G). Also, as an internal control for the specificity of the PLA reaction, only cells infected with EdU-KSHV (Fig. 3G, pink arrowhead) exhibited PLA signals, while uninfected cells (yellow arrowheads) did not.

Together, these data demonstrate that IFI16 interacts with KSHV LANA regardless of the presence of KSHV genome DNA, although the presence of KSHV genome DNA appears to stabilize this interaction further. The observation that a preformed IFI16-LANA complex colocalizes with the KSHV genome suggests that this IFI16-LANA complex is involved in one or more important functions that necessitate its binding to the KSHV genome DNA. However, this is beyond the scope of this article, and we are investigating this in a separate study.

### KSHV LANA acetylation increases upon lytic reactivation

**Fig 4:**
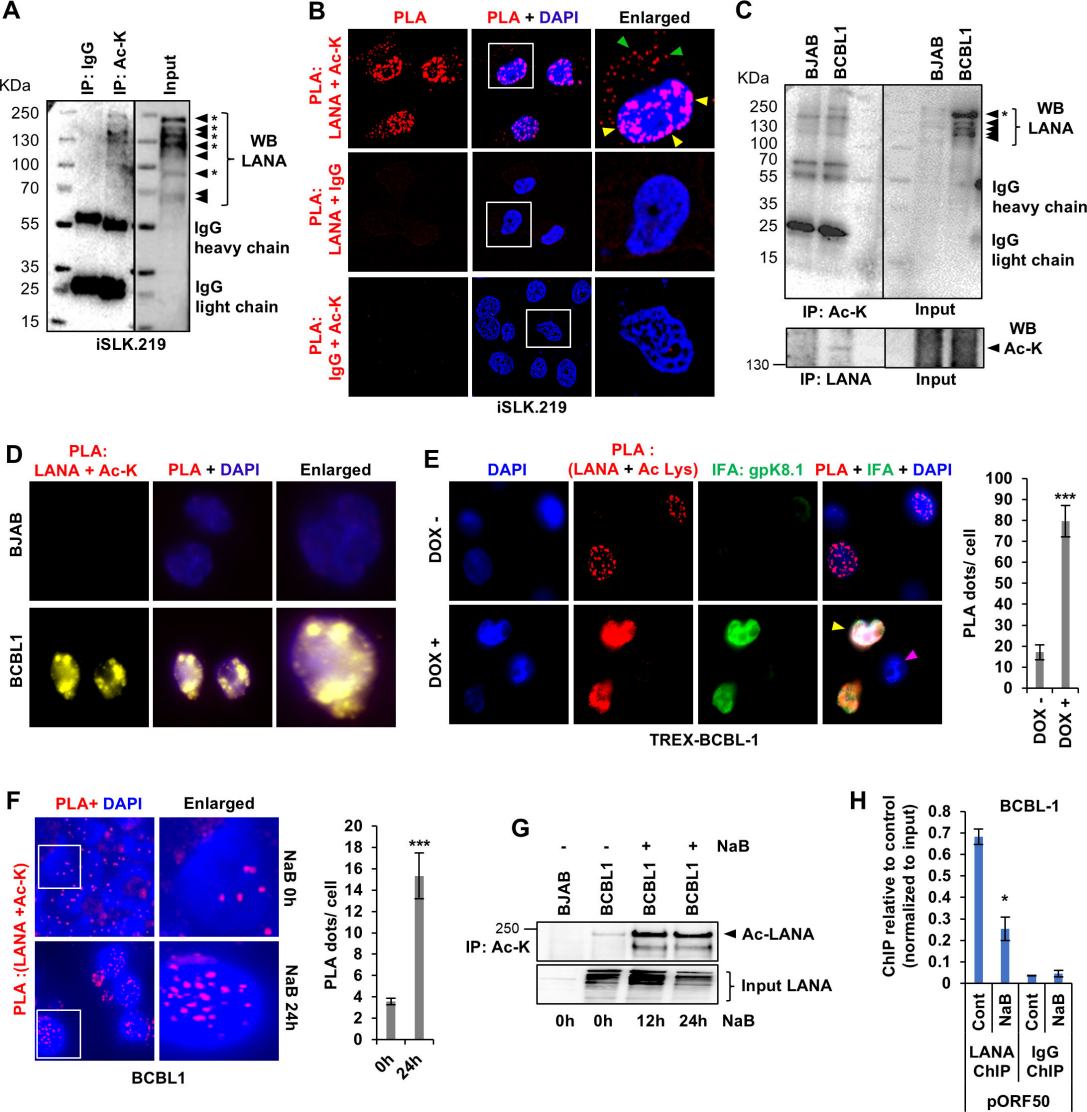
Acetylation/deacetylation of KSHV-LANA. (**A**) WCLs of KSHV(+) iSLK.219 cells were immunoprecipitated with anti-acetyl-K and IgG antibodies, and WB for LANA. The corresponding input exhibits different isoforms of LANA. The isoforms that were identified as acetylated are indicated with asterisks. (**B**) PLA (red) of LANA with acetyl-lysine (Ac-K) or isotype control IgG antibodies in iSLK.219 cells. Yellow arrowheads in the enlarged image point to the PLA dots inside the nucleus, while green arrowheads point to PLA signals in the cytoplasm. (**C**) WCLs of BJAB and BCBL1 cells were immunoprecipitated with the indicated antibodies and immunoblotted for LANA or Ac-K. (**D**) PLA (yellow) of LANA and Ac-K in BJAB and BCBL1 cells. (**E**) PLA (red) of LANA and Ac-K in uninduced (DOX–) and doxycycline-induced (DOX+, 24 h) TREX-BCBL1 cells. IFA (green) of the KSHV late lytic glycoprotein gpK8.1A is used to distinguish cells that are undergoing lytic reactivation (yellow arrowhead) from those that are not (pink arrowhead). The quantification of PLA dots per cell was conducted across three independent experiments, and the mean value was subsequently plotted for analysis. (**F**) PLA (red) of LANA and Ac-K in BCBL1 cells treated or untreated with sodium butyrate (NaB, 1 mM, 24 h). The quantification of PLA dots per cell was conducted across three independent experiments, and the mean value was subsequently plotted for analysis. (**G**) WCLs of BJAB and BCBL1 cells untreated or treated with NaB (12 and 24 h) were immunoprecipitated with anti-Ac-K antibody and WB for LANA. (**H**) ChIP analysis of BCBL1 cells after NaB treatment (12 h). Nuclear lysates were chromatin immunoprecipitated (ChIP) with anti-LANA or its isotype control antibodies, and the coimmunoprecipitated chromatin was analyzed by qPCR using primers specific to the ORF50 promoter region (pORF50). Relative promoter occupancy of LANA is presented in comparison between treated and untreated cells. Results are represented as mean ± SD of data from three independent experiments, and “*” denotes *P* < 0.05 (unpaired *t*-test).

KSHV LANA has previously been reported to undergo acetylation (47). Therefore, we next studied LANA acetylation by subjecting iSLK.219 cell lysates to IP using an anti-acetyl-lysine (Ac-K) antibody or its isotype control IgG. WB of the immunoprecipitated proteins with anti-LANA antibody revealed that among the eight LANA isoforms in the input, five were detectable as acetylated (Fig. 4A, indicated with asterisks). PLA using anti-LANA and anti-Ac-K also yielded strong signals in the nucleus of iSLK.219 cells (Fig. 4B, yellow arrowheads), with some signal in the cytoplasm (green arrowheads). Although the presence (71) and accumulation of LANA in the cytoplasm post-lytic reactivation (69) have been reported previously, the function of its acetylated isoforms in the cytoplasm has not been deciphered yet. Similarly, IP and PLA in BCBL1 cells also demonstrated the presence of acetylated LANA (Ac-LANA) in uninduced latent PEL cells (Fig. 4C and D, respectively). However, in contrast with iSLK.219 cells (Fig. 4A), in BCBL1 cells (Fig. 4C), we observed only one acetylated form of LANA corresponding to the full-length isoform (indicated with an asterisk).

Following this, we investigated the effect of lytic reactivation on the LANA acetylation. We reactivated TREX-BCBL1 cells with DOX and performed PLA for LANA and Ac-K, along with IFA against the KSHV late lytic protein gpK8.1A to identify cells undergoing reactivation. We observed that in cells that underwent lytic reactivation (Fig. 4E, yellow arrowhead), the PLA signal, i.e., Ac-LANA, was significantly higher than in cells that did not undergo reactivation (pink arrowhead) or were not treated with doxycycline (DOX–). A similar increase in LANA-Ac-K PLA signal was observed upon treating BCBL1 cells with 1 mM sodium butyrate (NaB), a class-I and class-IIa HDAC inhibitor that is also used for lytic reactivation of latently infected cells (72, 73) (Fig. 4F). IP with Ac-K antibody in BCBL1 cells treated with NaB also confirmed this observation and showed that by 12 h post-NaB treatment, acetylation of LANA increases significantly compared to untreated control (Fig. 4G).

Next, we investigated how LANA acetylation affects its ability to bind to the ORF50 promoter. For this, we performed ChIP on BCBL1 cells with or without NaB treatment. Our findings confirmed that NaB treatment results in the reduced binding of LANA to the ORF50 (RTA) promoter (pORF50) (Fig. 4H), implying that upon acetylation, LANA loses its ability to bind to the KSHV immediate-early RTA promoter. Together, these data confirm that KSHV LANA undergoes an acetylation/deacetylation cycle. The deacetylated form, which is capable of binding to the ORF50 promoter, predominates during prolonged latency, while the acetylated form, which is incapable of RTA-promoter binding, increases upon lytic reactivation.

### HDAC1 and HDAC2 deacetylate LANA

**Fig 5:**
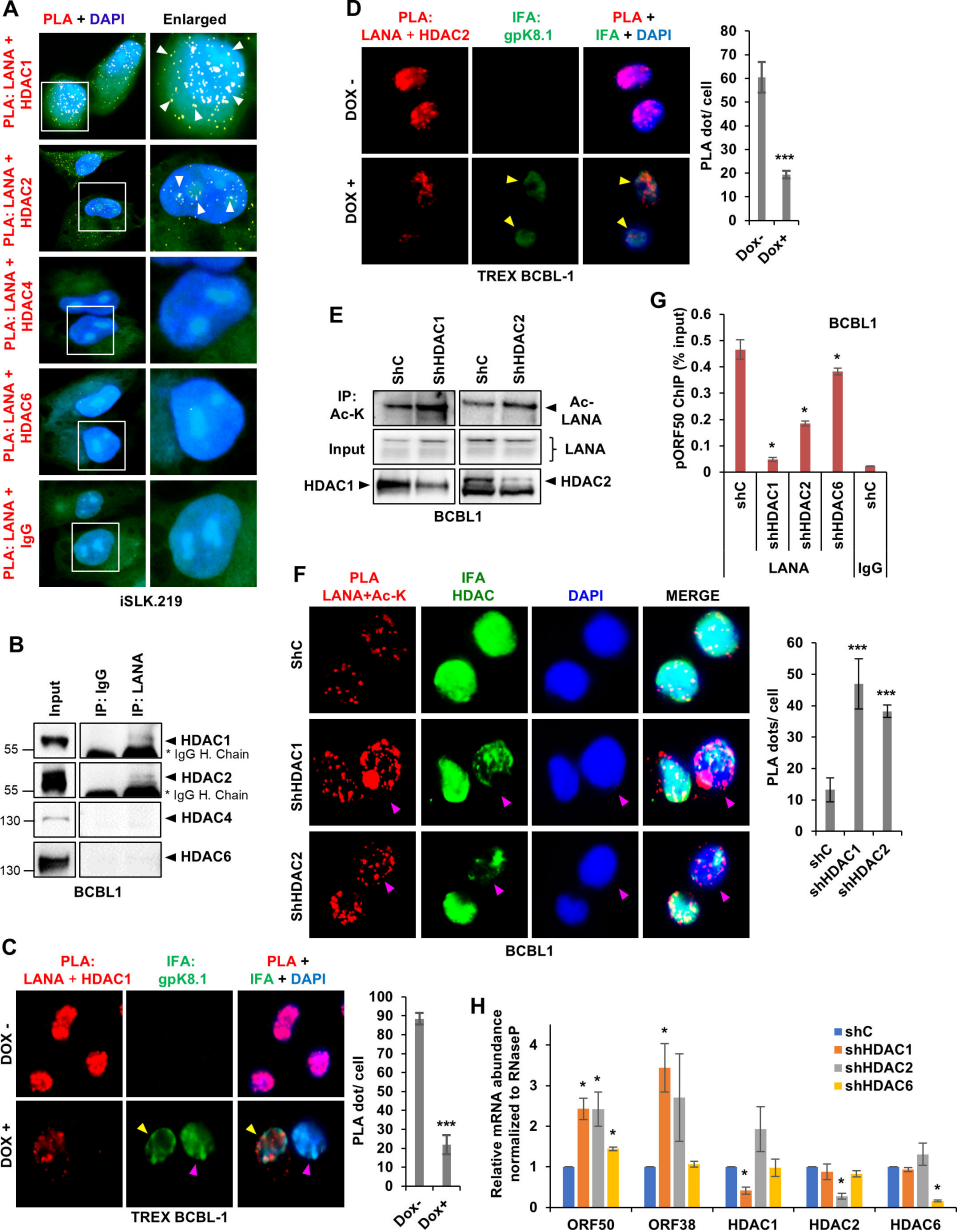
Role of HDAC1/2 in LANA acetylation. (**A**) PLA (yellow) of LANA with HDAC1, HDAC2, HDAC6, and IgG in iSLK.219 cells. (**B**) WCLs of BCBL1 cells were immunoprecipitated with anti-LANA and anti-IgG antibodies, and WB with HDAC1, HDAC2, HDAC4, and HDAC6 antibodies. (**C**) PLA (red) of LANA and HDAC1 in uninduced and doxycycline-induced (24 h) TREX-BCBL1 cells. IFA (green) of gpK8.1A confirms lytic cycle induction. Yellow arrows indicate induced cells, while the pink arrow highlights a cell that is significantly more induced. The quantification of PLA dots per cell was conducted across three independent experiments, and the mean value was subsequently plotted for analysis. (**D**) Similar to (C), PLA of LANA and HDAC2. (**E**) WCLs of control, HDAC1, and HDAC2 knockdown (KD) BCBL1 cells were immunoprecipitated with anti-acetyl-K antibody and WB with LANA. (**F**) PLA (red) of LANA acetylation using anti-LANA and anti-acetyl-K antibodies in control and HDAC1/2 KD BCBL1 cells. IFA (green) of HDAC1 and HDAC2 was performed to identify KD cells. Pink arrows identify cells in which HDACs have been efficiently knocked down. The quantification of PLA dots per cell was conducted across three independent experiments, and the mean value was subsequently plotted for analysis. (**G**) BCBl1 cells were transduced with shC, shHDAC1, shHDAC2, and shHDAC6 lentivirus, and 72 h later, LANA’s recruitment on the ORF50 promoter was evaluated by ChIP using anti-LANA and anti-IgG antibodies from the nuclear lysates. Results are represented as mean ± SD of data from three independent experiments, and “*” denotes *P* < 0.05 (unpaired *t*-test). (**H**) Total RNA was isolated from BCBL1 cells 72 h after HDAC1 KD. qRT-PCR was performed using gene-specific primers for HDAC1, HDAC2, KSHV late gene ORF25, and KSHV immediate-early gene ORF50. mRNA levels were normalized to β-actin mRNA levels and graphically represented as relative amounts compared to shC (control). Results are represented as mean ± SD of data from three independent experiments, and “*” denotes *P* < 0.05 (unpaired *t*-test).

Next, we verified that the class-I deacetylases HDAC1, and HDAC2 deacetylates KSHV LANA. To do this, we first studied LANA’s interaction with HDAC1, -2, -4, and -6 using PLA in iSLK.219 cells (Fig. 5A) and IP in BCBL1 cells (Fig. 5B). In both these latent cell lines, LANA was found to interact with HDAC1 and HDAC2 but not with HDACs 4 or 6. Subsequently, we studied the interactions between LANA and HDAC after lytic reactivation. For this, we performed PLA after reactivating TREX-BCBL1 cells with DOX for 24 h and included gpK8.1A IFA to identify reactivated cells. The results showed that both LANA-HDAC1 PLA (Fig. 5C) and LANA-HDAC2 PLA (Fig. 5D) signals were reduced after lytic reactivation (cells indicated by yellow arrowheads), implying that the LANA–HDAC1/2 interaction is a regulated event, which is determined by the phase of the viral life cycle. Interestingly, our observations also revealed that the interaction between LANA and HDAC1 is sensitive to the extent of lytic reactivation. Cells with higher expression of gpK8.1A, indicating more reactivation (Fig. 5C, cell indicated by the pink arrowhead), show almost no interaction between LANA and HDAC1 compared to cells with lower expression of gpK8.1A (Fig. 5C, yellow arrowhead). This observation is consistent with our previous finding (Fig. 4E) that the levels of Ac-LANA increase after lytic reactivation.

Following this, we evaluated the effects of HDAC1/2 depletion on LANA acetylation. IP using an Ac-K antibody showed that Ac-LANA levels increased after shRNA-mediated KD of HDAC1 and 2 in BCBL1 cells (Fig. 5E). Efficient KD of HDACs was confirmed by their respective WBs. In a similar experiment, using LANA + Ac-K PLA and IFA against the respective HDAC, we observed that compared to shC, shHDAC1- and 2-treated cells exhibited increased PLA signals (Fig. 5F). Particularly, cells with more pronounced KD of HDAC1 or HDAC2 (indicated by pink arrowheads) exhibited more significant elevation of Ac-LANA.

Next, we assessed the change in LANA’s binding to the pORF50 after KD of HDAC1/2 in BCBL1 cells. LANA ChIP showed that LANA’s binding on the pORF50 decreased by 9.6-fold upon HDAC1 depletion and by 2.5-fold after HDAC2 KD (Fig. 5G). In contrast, KD of HDAC6 resulted in only a 1.2-fold change, confirming that it does not play a role in LANA’s recruitment on pORF50. IgG ChIP was performed to confirm the specificity of the LANA ChIP (Fig. 5G). In the same experiment, we also evaluated the relative abundances of the ORF50 and the late lytic ORF38 mRNAs. We found that KD of HDAC1 and 2 resulted in a significant increase in their levels, while KD of HDAC6 had little to no effect (Fig. 5H). Efficient and specific KDs of HDAC1, HDAC2, and HDAC6 were confirmed by their relative mRNA levels in the same experiment (Fig. 5H).

These experiments establish that HDAC1/2 bind to KSHV LANA during latency, leading to its deacetylation. This deacetylated LANA binds to the RTA promoter and represses its transcription. Upon lytic reactivation, HDAC1/2 dissociates from LANA, leading to the accumulation of acetylated forms of LANA that have lesser affinity for the LANA-repressed RTA promoter. This leads to its transcriptional upregulation and the overall induction of the lytic cycle.

### IFI16-HDAC1/2-LANA forms a tripartite complex

**Fig 6:**
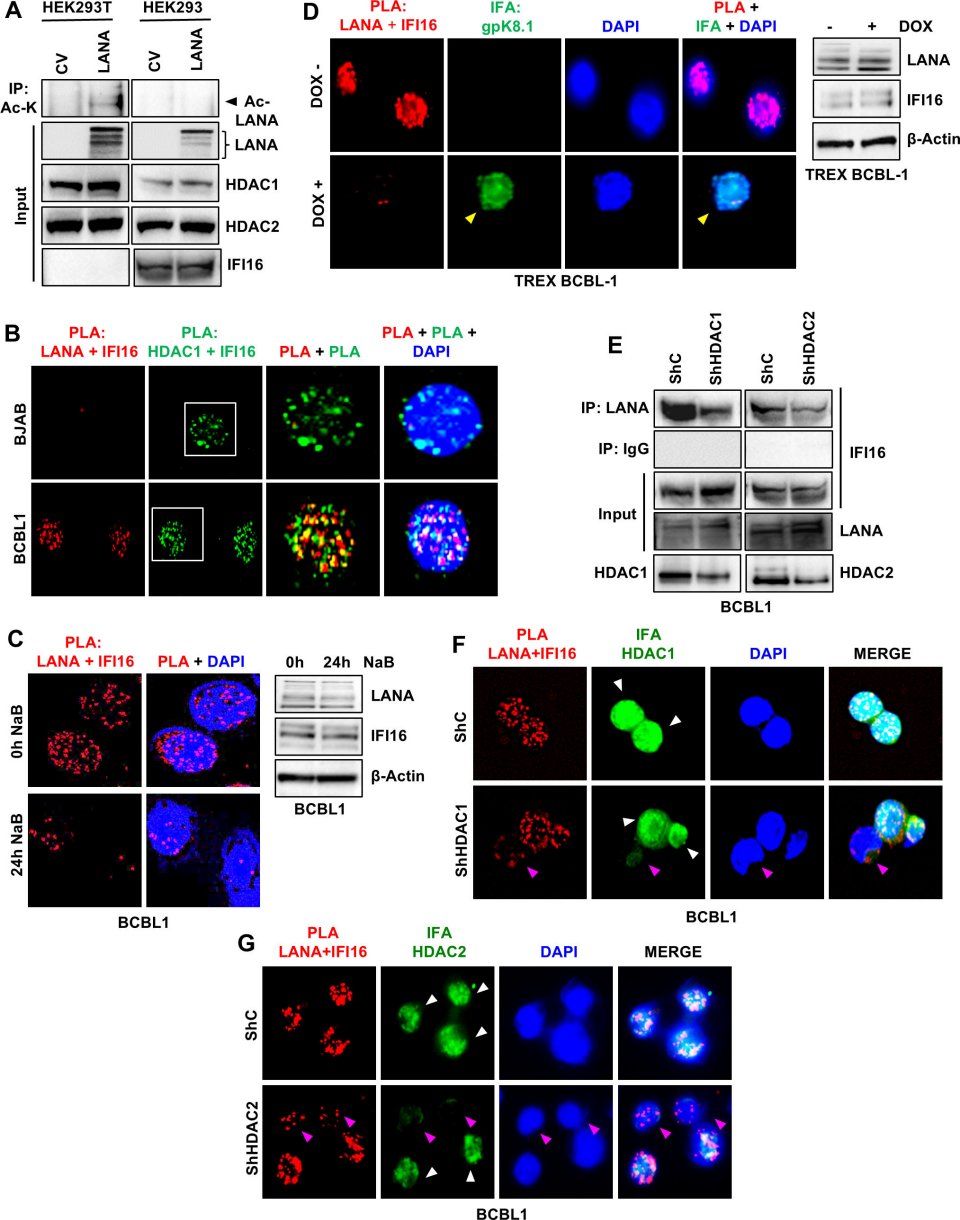
Formation of a tripartite complex by LANA, IFI16, and HDAC1/2. (**A**) WCLs of LANA transfected into HEK293T (IFI16 absent) and HEK-293 (IFI16 present) cells were immunoprecipitated with anti-acetyl-K antibody and immunoblotted with anti-LANA antibody. The presence of LANA, HDAC1, HDAC2, and IFI16 was confirmed by WB. (**B**) BJAB and BCBL1 cells were subjected to double PLA. First, a PLA (red) of LANA and IFI16 was performed, and then a second PLA (green) of HDAC1 and IFI16 was performed on the same cells. In the overlapped image of both the PLAs, yellow (pointed with white arrowheads) represents the interaction of the three proteins, LANA, IFI16, and HDAC1. (**C**) PLA of LANA and IFI16 in untreated and NaB-treated (24 h, 1 mM) BCBL1 cells. WB for LANA and IFI16 is also shown under these conditions. (**D**) PLA (red) of LANA and IFI16 in uninduced and doxycycline-induced (24 h) TREX-BCBL1 cells. IFA (green) of gpK8.1A confirms induction. The yellow arrowhead shows an induced cell. WB for LANA and IFI16 is also shown under these conditions. (**E**) WCLs of control KD (shC) and HDAC1/2 KD BCBL1 cells were immunoprecipitated with anti-LANA or anti-IgG antibodies, and WB for IFI16. Efficient KDs of HDAC1/2 are also shown. (**F and G**) PLA (red) of LANA and IFI16 in HDAC1/2 KD or control BCBL1 cells. KD of HDAC1/2 is shown by IFA (green). Cells exhibiting efficient KD are pointed with pink arrowheads, while white arrowheads point to cells that did not undergo KD or were treated with shC.

Based on our observations described so far (Fig. 1 to 5), we next hypothesized that IFI16 recruits HDAC1/2 to LANA during latency, helping to maintain it in a deacetylated state that is competent to repress lytic transcription and reactivation effectively. This hypothesis was strengthened by our next observation that when LANA is transfected into HEK293T and HEK293 cells, Ac-LANA is detectable in HEK293T but not in HEK293 (Fig. 6A). Both cell types express HDAC1/2, but while HEK293 cells naturally express IFI16, HEK293T cells lack IFI16 expression. To further validate our hypothesis, we performed a double PLA experiment: LANA + IFI16 PLA (red) and IFI16 + HDAC1 PLA (green) in tandem. The results showed that both PLA signals extensively colocalized with each other (Fig. 6B, white arrowheads), indicating that these three proteins form a tripartite complex. Next, we observed that when the HDAC1/2 activity was inhibited by NaB in BCBL1 cells, the interaction between IFI16 and LANA was reduced (Fig. 6C). Consistent with this finding and our previous observation (Fig. 4E) that Ac-LANA levels increase after lytic reactivation, we also observed that inducing lytic reactivation in TREX-BCBL1 cells (expressing gpK8.1A, indicated by the yellow arrowhead) led to a decreased interaction between IFI16 and LANA (Fig. 6D).

Following this, we investigated whether HDAC1 and HDAC2 are essential components of the IFI16-HDAC-LANA tripartite complex, crucial for its stability. To do this, we silenced HDAC1/2 in BCBL1 cells and examined the interaction between LANA and IFI16 using IP. Silencing both HDACs led to a notable decrease in the IP of IFI16 by anti-LANA antibodies (Fig. 6E). In a similar experiment, we compared the LANA + IFI16 PLA in HDAC KD cells to control KD cells. These cells were also stained for HDAC1/2 to help identify cells that underwent efficient KD. We found that KD of both HDAC1 and HDAC2 resulted in reduced IFI16-LANA PLA signals (Fig. 6F and G, cells highlighted in pink arrowheads). Thus, we conclude that IFI16, HDAC1/2, and LANA form a tripartite complex, with the acetylated form of LANA being the primary participant in this complex. Additionally, our findings also demonstrate that HDAC1 and HDAC2 are integral components of this complex and that their depletion leads to its destabilization.

### IFI16 recruits HDAC1/2 to LANA

**Fig 7:**
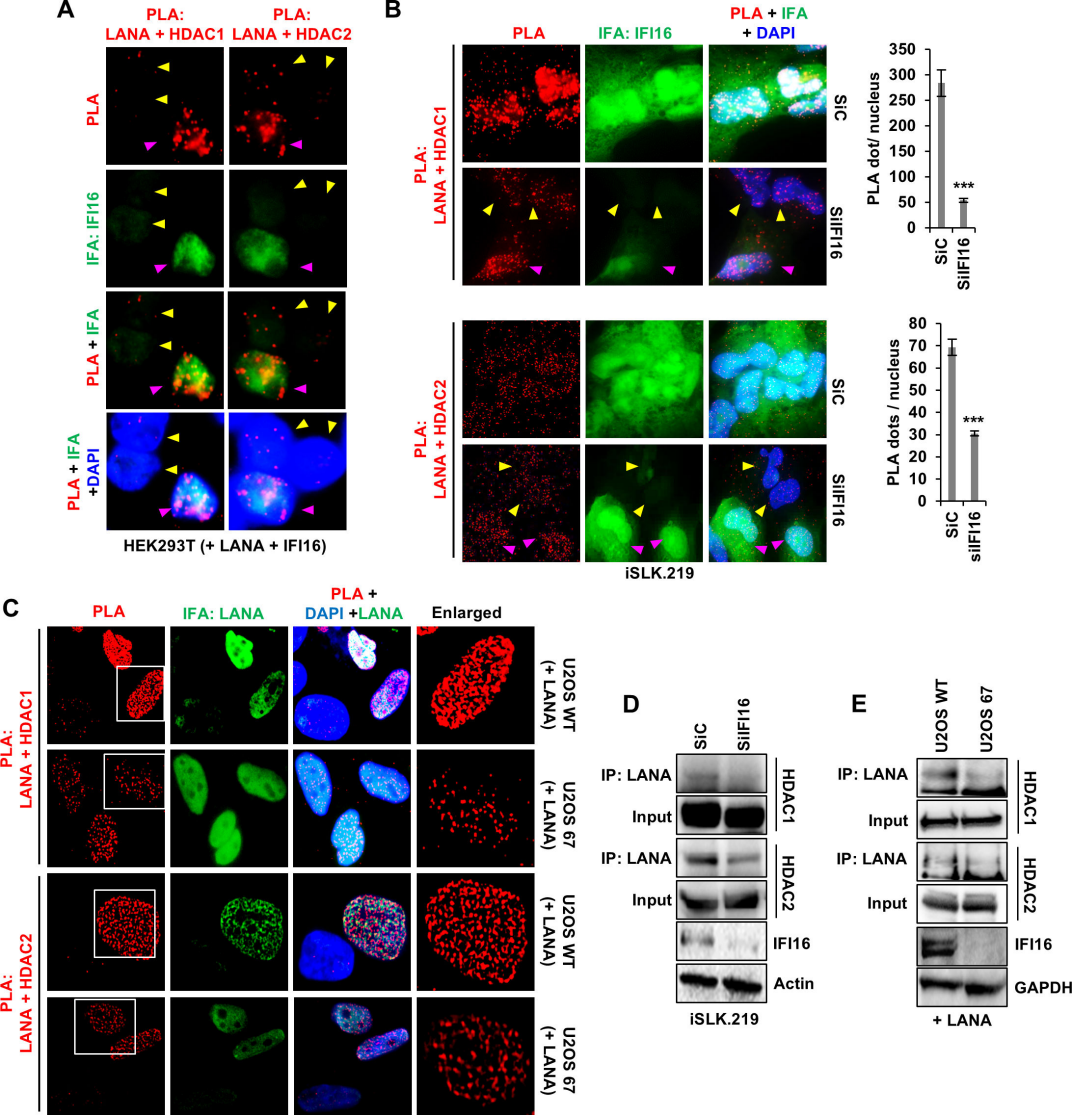
Importance of IFI16 for LANA and HDAC1/2 interaction. (**A**) PLA (red) for LANA with HDAC1 and HDAC2 in HEK293T cells transfected with LANA and IFI16-expressing plasmids. IFI16 plasmid was used at a 1/5 concentration as that of LANA. IFA (green) shows IFI16 expression. Yellow arrowheads point to cells lacking IFI16, while pink arrowheads point to IFI16-expressing cells. (**B**) PLA (red) of LANA with HDAC1 and HDAC2 in siC- and siIFI16-treated latent iSLK.219 cells. IFA (green) shows IFI16 expression. Yellow arrowheads point to cells in which IFI16 was efficiently knocked down, while pink arrowheads point to cells still IFI16 expressing. The quantification of PLA dots per cell was conducted across three independent experiments, and the mean value was subsequently plotted for analysis. (**C**) PLA of LANA with HDAC1 or HDAC2 in U2OS WT (IFI16 positive control cells) and U2OS 67 (IFI16 knockout) cells. IFA (green) shows LANA expression. (**D**) WCLs of control and IFI16 KD iSLK.219 cells (siRNA) were immunoprecipitated with anti-LANA and WB for HDAC1 and HDAC2. KD of IFI16 is also shown. (**E**) Similar to (D), LANA antibodies were used to pull down HDAC1/2 in IFI16 KO (U2OS 67) or wild-type U2OS cells.

In order to further support our hypothesis that IFI16 recruits HDAC1/2 to LANA, we transfected HEK293T cells with LANA and IFI16 expression plasmids. The IFI16 plasmid was used at a 1/5 concentration compared to the LANA plasmid, ensuring that while all cells received LANA, only a fraction of the cells received IFI16. We conducted PLA for LANA-HDAC1/2 and IFA for IFI16. The results showed that cells expressing IFI16 exhibited much higher levels of LANA-HDAC1/2 PLA signal (Fig. 7A, cells highlighted in pink arrowheads) compared to cells that did not receive IFI16 (cells highlighted in yellow arrowheads).

Next, we depleted IFI16 in iSLK.219 cells via siRNA and probed for LANA–HDAC1/2 interaction via PLA and IFI16 KD by IFA. We observed that cells that underwent efficient IFI16 KD (Fig. 7B, highlighted in yellow arrowheads) showed reduced LANA-HDAC1/2 PLA compared to cells that did not undergo efficient KD (Fig. 7B, highlighted in pink arrowheads) or cells treated with siC. Similar observations were also made in U2OS cells knocked out (KO) of IFI16 using CRISPR Cas9 (U2OS 67) (14). When transfected with LANA, compared to U2OS WT, U2OS 67 cells exhibited drastically low levels of LANA-HDAC1/2 PLA (Fig. 7C). LANA expression was confirmed by IFA (Fig. 7C). Co-IP between LANA and HDAC1/2 in IFI16 KD iSLK.219 cells (Fig. 7D) and IFI16 KO U2OS 67 cells (Fig. 7E) also showed similar results, where depletion of IFI16 resulted in reduced LANA–HDAC1/2 interactions, confirming that IFI16 is essential for the recruitment of HDAC1/2 on KSHV LANA.

### IFI16 drives LANA deacetylation during latency, facilitating its binding to the ORF50 promoter

**Fig 8:**
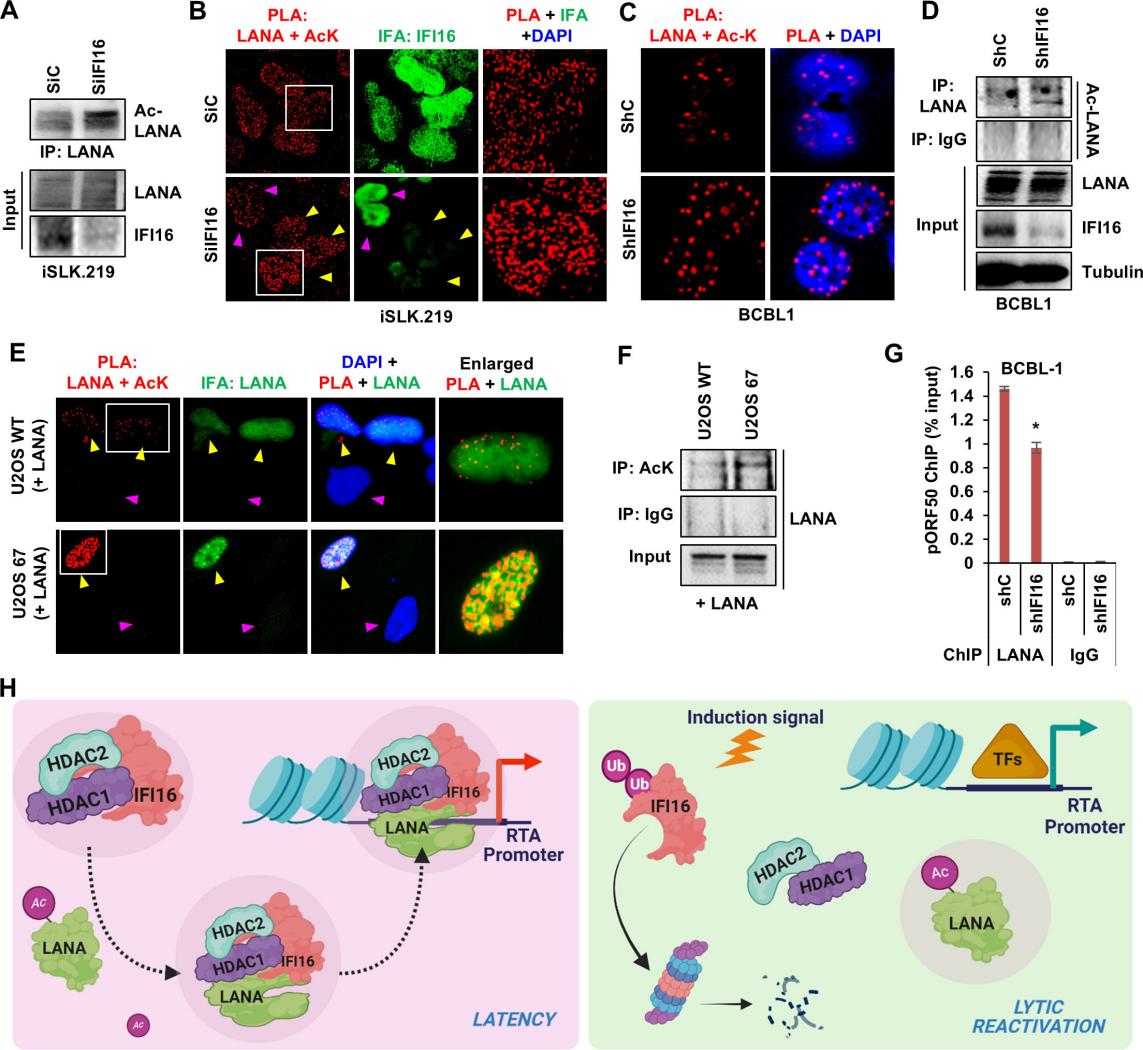
Role of IFI16 in LANA deacetylation. (**A**) WCLs of iSLK.219 cells treated with siC or siIFI16 were immunoprecipitated with anti-LANA antibodies and WB for acetyl-K. Input LANA and IFI16 KD are also shown. (**B**) PLA (red) for LANA and acetyl-K in control and IFI16 KD iSLK.219 cells. IFA (green) shows IFI16 expression. Yellow arrowheads point to cells in which IFI16 was knocked down, while pink arrowheads point to cells that did not undergo siRNA-mediated silencing of IFI16. (**C**) PLA (red) for LANA and acetyl-K in shC- and shIFI16-transduced BCBL1 cells. (**D**) WCLs of control and IFI16 KD BCBL1 cells were immunoprecipitated with anti-LANA and anti-IgG antibodies, and WB for acetyl-K. Input LANA and IFI16 KD are also shown. (**E**) PLA (red) for LANA and acetyl-K in LANA-transfected U2OS WT and U2OS 67 (IFI16 KO) cells. IFA (green) shows LANA. Yellow arrowheads point to cells that received LANA plasmid, while pink arrowheads point to cells that did not. (**F**) WCLs of LANA-transfected U2OS WT and U2OS 67 (IFI16 KO) cells were immunoprecipitated with anti-acetyl-K and anti-IgG antibodies, and WB for LANA. (**G**) Nuclear lysates of control and IFI16 KD BCBL1 cells were chromatin immunoprecipitated with anti-LANA and control IgG antibodies, and coimmunoprecipitated DNA was analyzed via qPCR using primers specific to the ORF50 promoter region. Relative promoter occupancy of LANA is presented in comparison between control and knockdown cells. Results are represented as mean ± SD of data from three independent experiments, and “*” denotes *P* < 0.05 (unpaired *t*-test). (**H**) Schematic representation of the IFI16-mediated recruitment of HDAC1/2, leading to LANA deacetylation in KSHV latency-lytic balance. LANA, by default, is post-translationally acetylated (Ac-LANA) by cellular lysine acetyltransferases (KATs). Although the specific KAT responsible for LANA acetylation has yet to be identified, our unpublished preliminary observations suggest a potential involvement of p300 in this process. In the nucleus, IFI16, which exists in a complex with the type I HDACs, HDAC1 and HDAC2, recruits these enzymes to Ac-LANA, facilitating its deacetylation. The resulting deacetylated form of LANA exhibits the ability to bind to the KSHV-RTA promoter, leading to the recruitment of various transcription factors (TFs) and, consequently, transcriptional repression (illustrated by the red arrow). Upon lytic induction, IFI16 undergoes degradation via the proteasomal pathway, causing the disassociation of HDAC1 and HDAC2 from LANA. This event results in the accumulation of Ac-LANA, which ultimately leads to the detachment of LANA from the RTA promoter. This disassociation triggers the expression of RTA (indicated by the green arrow) and subsequently initiates the downstream expression of additional lytic genes, thereby promoting KSHV lytic replication. Illustration created with BioRender.com.

Finally, to conclusively prove that the IFI16-mediated recruitment of HDAC1/2 leads to LANA deacetylation, we first evaluated Ac-LANA levels in IFI16 KD (siRNA) iSLK.219 cells. Commensurate with IFI16’s role in HDAC1/2 recruitment (Fig. 7), the depletion of IFI16 led to the hyperacetylation of LANA (Fig. 8A). Similar observations were also made by PLA (Fig. 8B), where control cells and cells that did not undergo IFI16 KD (pink arrowheads) exhibited lower LANA + Ac-K PLA signal compared to cells in which IFI16 was efficiently knocked down (KD) (yellow arrowheads). PLA and IP of Ac-LANA in control and IFI16 KD BCBL1 cells (shRNA) also show a significant increase in LANA acetylation in the IFI16 KD condition (Fig. 8C and D, respectively). In U2OS WT and 67 cells (IFI16 KO) transfected with LANA, LANA acetylation was drastically increased in U2OS 67 cells compared to WT (PLA, Fig. 8E, and IP, Fig. 8F). As a confirmation of the specificity of the PLA reaction, the presence of LANA + Ac-K PLA was only observed in cells positively transfected with LANA (IFA, Fig. 8E). Furthermore, LANA ChIP (Fig. 8G) confirmed that KD of IFI16 in BCBL1 cells results in the decreased binding of LANA to the pORF50, establishing IFI16’s role in regulating LANA promoter binding via its deacetylation.

## DISCUSSION

The balance between latency and the lytic cycle is a crucial factor in KSHV pathogenesis and tumorigenesis, and, like other herpesviruses, KSHV has evolved multiple mechanisms to tightly regulate this balance. In this study, we have identified a novel mechanism by which KSHV utilizes the physiological interaction between IFI16 and the class-I HDACs, HDAC1 and 2, to promote the deacetylation of its major latency protein, LANA, which, in turn, influences LANA’s ability to bind and silence the promoter of the KSHV lytic switch protein, RTA, thereby maintaining latency. This finding adds to our previously described role of IFI16 in maintaining KSHV latency, where we found that IFI16 recruits the H3K9me3 MTases, SUV39H1, and GLP to silence KSHV lytic genes epigenetically (27). We propose that these two mechanisms are mutually exclusive yet complementary, and both are crucial for latency establishment and maintenance.

LANA’s role as a critical regulator of the KSHV latent/lytic balance is well established. One of the most prominent ways in which LANA regulates the KSHV life cycle is by functioning as a transcription modulator of various KSHV promoters (35, 36, 45, 46, 48, 50). Multiple studies have reported LANA’s ability to bind to several KSHV gene promoters from all temporal gene classes and recruit various transcription and epigenetic factors on them (50, 52, 74). Among these, LANA’s binding to the ORF50 promoter is perhaps the most consequential, where it recruits transcription factors such as Sp1, RBP-J, Nrf2, and KAP1 to suppress the expression of the KSHV RTA, facilitating latency (47–49, 75). However, for LANA to effectively serve as a regulator of the KSHV latent/lytic switch, its binding to KSHV promoters must be regulatable, i.e., LANA must be able to dissociate from the lytic promoters during reactivation. Acetylation of LANA has been proposed to act as this regulatory switch, where the deacetylated form binds to the RTA promoter while the acetylated form does not. LANA acetylation was first reported in 2006 by Lu et al., where they found that HDAC inhibitors disrupt LANA’s interaction with Sp1 and histone H2B, and cause rapid dissociation of LANA from the RTA promoter (47). The specific lysine residues of LANA that undergo acetylation are currently unknown. Hellert et al. reported that the C-terminal domains of both KSHV LANA and MHV-68 LANA contain a characteristic lysine-rich positively charged surface patch, which appears to be a unique feature of γ2-herpesviral LANA proteins (40). This basic patch has been shown to contribute to LANA’s binding to the terminal repeat LBS and to the establishment of latency (39, 40). In addition, the ability of LANA to undergo higher-order oligomerization and form nuclear speckles has also been proposed to depend on this C-terminal lysine patch (40, 41). Whether this lysine patch plays a role in LANA’s binding to KSHV promoters and if one or multiple lysine residues in this patch undergo acetylation to alter LANA’s binding to KSHV promoters are questions beyond the scope of this article but are being currently investigated in our laboratory.

The process of lysine acetylation is well known for its important roles in protein subcellular localization, stability, and protein–protein and protein–DNA interactions (76–78). This report and previous studies confirm that acetylation of KSHV LANA affects its ability to bind to the RTA promoter. However, several questions on the role of LANA acetylation remain unaddressed. (i) Does acetylation affect LANA’s stability and subcellular localization? (ii) Does acetylation affect LANA’s interaction with other viral or cellular proteins? (iii) Does acetylation impact LANA’s other critical functions, such as KSHV DNA replication and metaphase chromosome attachment?

The specific lysine acetyltransferase (KAT) that acetylates LANA has not been elucidated yet; however, there are reports that LANA interacts with the KATs, CREB-binding protein (CBP), and p300 (79). We are currently investigating the role of these KATs in LANA acetylation. In regard to lysine deacetylases (KDACs), although LANA has been previously shown to interact with HDAC1 and HDAC2 (80, 81), the evidence presented in this and previous reports suggests that LANA is unable to interact with these KDACs on its own and needs a recruiter protein to do so (81). Here, we have identified nuclear IFI16 as a recruiter of HDAC1/2 on KSHV LANA. IFI16 has been previously shown to bind several nuclear proteins, including RIG-I, P53, SP1, BRCA1, ADAR1, APOBEC3B, BRD4, DDX10, SUV39H1, GLP, etc. (25, 61, 62, 67, 82–84). The PYRIN domain of IFI16 known to facilitate protein–protein interaction (85) has been elucidated to play a role in some of these interactions. The novel interaction between IFI16 and the class-I HDACs, HDAC1 and 2, identified in this study, adds to this repertoire of IFI16 interacting proteins. It is evident from the numerous interactions of IFI16 that it is a multifunctional protein. Although the physiological significance of the IFI16–HDAC1/2 interaction identified here is currently unknown, it is reasonable to hypothesize that IFI16 may also be involved in recruiting HDAC1/2 to the KSHV chromatin to epigenetically regulate its expression. If proven, this will add to the previously described epigenetic role of IFI16, where we found that IFI16 binds and recruits the histone methyltransferases, SUV39H1 and GLP, on the KSHV chromatin (25).

Sarek et al. demonstrated that the interaction between LANA and HDAC1 is dependent on the presence of nucleophosmin (NPM), a nucleolar histone chaperone (81). They showed that NPM binds HDAC1, and this interaction is necessary for KSHV latency. Interestingly, in our LC-MS-MS evaluation of IFI16-associated cellular proteins (Fig. 1A), we detected NPM as a high-confident interactor of IFI16 in PEL cells (data not shown). This raises the possibility that IFI16 and NPM together form an HDCA1/2 recruiting complex that facilitates the deacetylation of LANA. Additional research is needed to explore this possibility, which is beyond the scope of this article. However, IFI16’s ability to form multi-protein macromolecular complexes has been proposed before (15, 86).

We have previously reported that lytic reactivation of cells latently infected with KSHV leads to the ubiquitination of IFI16, followed by its degradation via the proteasomal pathway (28). We propose that this depletion of IFI16 after lytic reactivation relieves the recruitment of HDACs on LANA, leading to the accumulation of acetylated LANA. According to this model (Fig. 8H), IFI16, at least a fraction of it, physiologically exists in complex with HDAC1/2 in the nucleus. Post-KSHV infection, when LANA is expressed, by default, it is post-translationally acetylated by yet unidentified KATs to form Ac-LANA. IFI16 binds this Ac-LANA and recruits HDAC1/2 to deacetylate it, shifting the equilibrium toward deacetylated LANA. This deacetylated LANA binds to the KSHV RTA promoter and possibly other KSHV promoters, and drives their silencing by recruiting transcriptional repressors and epigenetic modifiers, establishing latency. Latency is maintained as long as the equilibrium between LANA and Ac-LANA favors LANA. Upon lytic induction, IFI16 undergoes targeted degradation, leading to the de-recruitment of HDAC1/2 from LANA, resulting in the accumulation of Ac-LANA. Ac-LANA detaches from the KSHV promotors, relieving them of the repressive pressure, allowing their expression, and initiating the downstream lytic reactivation cascade. Together, this study enhances the current understanding of KSHV latency regulation and uncovers a new role of IFI16 in this process.
